# Long-Term Outcomes of Epidural Motor Cortex Stimulation for Refractory Chronic Neuropathic Orofacial Pain

**DOI:** 10.3390/life16040651

**Published:** 2026-04-12

**Authors:** Marina Raguž, Marko Tarle, Petar Marčinković, Sven Krušić, Domagoj Dlaka, Tonko Marinović, Darko Chudy

**Affiliations:** 1 Department of Neurosurgery, Dubrava University Hospital, 10000 Zagreb, Croatia; petar.marcinkovic11@gmail.com (P.M.); skrusic@kbd.hr (S.K.); domagojdlaka@gmail.com (D.D.); tmarinovic@kbd.hr (T.M.); darko.chudy@gmail.com (D.C.); 2 School of Medicine, Catholic University of Croatia, 10000 Zagreb, Croatia; 3 Department of Maxillofacial Surgery, Dubrava University Hospital, 10000 Zagreb, Croatia; mtarle@sfzg.hr; 4 School of Dental Medicine, University of Zagreb, 10000 Zagreb, Croatia; 5 Medicine of Sports and Exercise Chair, Faculty of Kinesiology, University of Zagreb, 10000 Zagreb, Croatia; 6 School of Medicine, University of Zagreb, 10000 Zagreb, Croatia

**Keywords:** motor cortex stimulation, neuropathic orofacial pain, chronic neuropathic pain, invasive neuromodulation, refractory pain, long-term outcomes, stimulation dependency

## Abstract

Background: Epidural motor cortex stimulation (MCS) is an established neuromodulatory option for refractory neuropathic pain; however, structured data on long-term outcomes, stimulation dependency, and real-world device management remain limited, particularly in chronic neuropathic orofacial pain. Methods: This retrospective single-center cohort study included patients with refractory neuropathic orofacial pain treated with epidural MCS at a tertiary neurosurgical center. Clinical data were extracted from medical records and longitudinal follow-up documentation. Pain intensity was assessed using a unified 0–10 numerical rating scale (NRS/VAS) at baseline, best achieved response, and last follow-up. Responder status was defined at the last follow-up (≥50% pain reduction from baseline). Secondary outcomes included stimulation dependency during OFF periods, reprogramming burden, device-related events, and safety. Results: Ten patients (6 women, 4 men; mean age 61.5 ± 8.6 years) were followed for a mean of 7.6 ± 6.3 years (range 2–22 years), with 70% exceeding five years of follow-up. Baseline pain intensity (8.8 ± 0.4) decreased to 4.6 ± 0.8 at the best achieved response and remained lower at last follow-up (5.6 ± 0.9). At the last follow-up, eight patients (80%) were classified as partial responders (30–49% pain reduction), while two (20%) were classified as non-responders. Clinically relevant worsening during stimulator OFF periods occurred in 70% of patients. Reprogramming was required in all patients, and 60% underwent battery replacement. No clinically significant stimulation-related adverse effects were observed. Conclusions: Epidural MCS was associated with sustained pain reduction over extended follow-up. These findings support the interpretation of MCS as a chronic neuromodulatory therapy requiring ongoing stimulation, individualized programming, and long-term device management, contributing clinically relevant long-term evidence to the evolving role of neuromodulation in refractory chronic neuropathic pain.

## 1. Introduction

Chronic pain represents a major global health burden and is increasingly conceptualized as a heterogeneous condition encompassing distinct pathophysiological mechanisms rather than a single clinical entity. Neuropathic pain represents a particularly challenging subtype due to its complex neurobiology, frequent treatment resistance, and substantial impact on long-term patient functioning. Contemporary frameworks differentiate chronic pain into nociceptive, neuropathic, and nociplastic categories, reflecting differences in underlying neurobiology and therapeutic responsiveness [1,2]. Neuropathic pain, defined as pain arising as a direct consequence of a lesion or disease affecting the somatosensory nervous system, remains particularly challenging to manage, especially when involving trigeminal and orofacial territories [3]. Neuropathic orofacial pain comprises a heterogeneous group of disorders, including post-traumatic trigeminal neuropathy, post-herpetic neuralgia, anesthesia dolorosa, and centrally mediated facial pain syndromes. These conditions are frequently refractory to first-line pharmacological therapies such as anticonvulsants and antidepressants and are associated with substantial impairment in quality of life and psychosocial functioning [4,5]. Even interventional procedures targeting peripheral or cranial nerve structures may yield an incomplete or transient benefit, underscoring the importance of addressing central pain-processing mechanisms in selected patients [6,7].

Motor cortex stimulation (MCS) has emerged as an invasive neuromodulatory option for patients with refractory neuropathic pain who have exhausted conservative and less invasive treatments. Following the pioneering observations by Tsubokawa and colleagues in central post-stroke pain [8,9], MCS has been applied to a range of neuropathic pain syndromes, including trigeminal and orofacial pain. Rather than directly interrupting nociceptive transmission, MCS is believed to modulate distributed pain-processing networks involving cortico-thalamic and cortico-limbic circuits [10,11,12]. Functional imaging and neurophysiological investigations support the concept that motor cortex stimulation influences both sensory-discriminative and affective-motivational dimensions of pain [10,11,13].

Recent systematic analyses highlight both the potential analgesic effects and the persistent methodological limitations of the current MCS literature. A systematic review and meta-analysis by Henssen et al. [14] emphasized the need for heterogeneity in patient selection, outcome reporting, and follow-up duration, as well as standardized definitions and longer-term data. Similarly, contemporary narrative reviews have noted that while meaningful analgesic effects are reported across neuropathic pain etiologies, evidence remains limited by small sample sizes and methodological variability [15]. Controlled data, although scarce, suggest that active cortical stimulation may provide greater analgesic benefit than sham conditions in selected patients [16], further supporting a biological basis for the observed effects.

Despite over two decades of clinical application, most published MCS series focus on short- to mid-term outcomes, often limited to 6–24 months of follow-up, with inconsistent definitions of clinical response [12,17]. In neuropathic orofacial pain, where disease trajectories may evolve over years, such time frames may not fully capture the durability of benefits or the need for ongoing device management. Consequently, structured long-term clinical data addressing the durability of analgesia, stimulation dependency, and real-world device management remain limited. Detailed reporting of long-term reprogramming requirements, medication adjustments, device-related events, and potential attenuation of the analgesic effect remains limited [18,19,20]. From a clinical perspective, MCS may be more appropriately considered a chronic neuromodulatory therapy requiring sustained follow-up and individualized optimization rather than a single procedural intervention. Understanding long-term pain trajectories, stimulation dependency, and adaptive programming strategies is, therefore, essential for realistic patient counseling and treatment planning within contemporary mechanism-informed chronic pain care models [11,21].

The present study aimed to evaluate long-term clinical outcomes and management characteristics of epidural MCS in patients with refractory neuropathic orofacial pain treated at a tertiary referral center. By systematically analyzing longitudinal pain trajectories, durability of response, stimulation reprogramming patterns, medication changes, and device-related events over extended follow-up, this study aims to contribute structured long-term clinical data to the evolving literature on invasive cortical neuromodulation for chronic neuropathic pain.

## 2. Materials and Methods

In this retrospective study, we analyzed data from electronic medical records of the Department of Neurosurgery, Referral Centre for Stereotactic and Functional Neurosurgery, Dubrava University Hospital, Zagreb, Croatia, between 1 January 2010 and 31 December 2025. Clinical data were collected through a systematic review of electronic medical records, operative reports, and longitudinal follow-up documentation. We included 10 consecutive patients admitted and treated using epidural MCS. Epidural paddle electrodes were implanted over the primary motor cortex contralateral to the dominant pain distribution, targeting the facial representation area of the motor homunculus. Cortical targeting was based on preoperative neuroimaging and anatomical landmarks, and confirmed intraoperatively using neuronavigation and/or neurophysiological mapping when applicable. The electrode was secured to minimize migration, and the implantable pulse generator (IPG) was placed subcutaneously according to institutional practice. Postoperative imaging was obtained in all cases to confirm correct electrode positioning. Device technology evolved during the study period, including the use of both non-rechargeable and rechargeable implantable pulse generators. In patients with longer follow-up, system replacement or upgrade to newer-generation devices was performed when clinically indicated, most commonly at the time of battery depletion.

The study was conducted in accordance with the Declaration of Helsinki and approved by the institutional ethics committee. Given the retrospective design and the use of fully anonymized clinical data, informed consent for participation was waived in accordance with institutional and national regulations.

Patients were eligible for inclusion if they met all of the following criteria: (a) diagnosis of chronic neuropathic orofacial pain according to International Association for the Study of Pain (IASP) criteria [1,2,3]; (b) pain refractory to optimized pharmacological treatment and prior interventional or surgical therapies, as documented in medical records; (c) treatment with epidural MCS targeting the primary motor cortex; and (d) availability of clinical follow-up data for a minimum of 12 months after implantation. Patients were excluded if MCS was implanted for indications other than neuropathic orofacial pain or if pain syndromes were classified as predominantly nociceptive or nociplastic without a clear neuropathic component. Patients treated with alternative neuromodulation targets (e.g., deep brain stimulation) were also excluded.

Patients were evaluated within a multidisciplinary clinical framework, including neurosurgery, neurology, and maxillofacial specialists. Psychological assessment was performed when clinically indicated as part of the preoperative evaluation. The diagnosis of neuropathic orofacial pain was established according to IASP criteria based on a combination of clinical history, neurological examination, and available imaging studies. Particular attention was given to the identification of a relevant lesion or disease affecting the trigeminal or related somatosensory pathways. Central versus peripheral etiologies were classified based on the clinical history, imaging findings, underlying cause, and anatomical level of the lesion. Post-surgical and post-traumatic cases were primarily considered peripheral neuropathic pain, whereas pain associated with cerebrovascular injury was classified as central neuropathic pain. In cases with overlapping features, classification was based on the predominant clinical and etiological component. All patients underwent extensive preoperative evaluation and were considered for MCS only after failure of optimized pharmacological therapy (including anticonvulsants and antidepressants) and prior interventional or surgical treatments, when applicable. The selection pathway followed a stepwise clinical approach, including confirmation of neuropathic pain according to IASP criteria, failure of optimized pharmacological and interventional treatments, and multidisciplinary evaluation prior to consideration of invasive neuromodulation. The decision to proceed with MCS implantation was made on an individual basis within a multidisciplinary team, taking into account pain severity, functional impairment, and lack of response to previous therapies. Trigeminal pain distribution was classified as mixed (V1/V2/V3) in all patients, reflecting the clinically diffuse and overlapping nature of neuropathic pain in the orofacial region, where strict dermatomal boundaries are often not observed.

Initial stimulation programming followed a conservative titration strategy, with gradual adjustment of stimulation parameters based on clinical response and patient tolerability. Parameters adjusted included amplitude, pulse width, and frequency. Across the cohort, stimulation amplitude ranged between 2 and 8 (mA or V, depending on constant-current vs. constant-voltage device configuration), pulse width ranged between 60 and 180 μs, while frequency ranged between 40 and 110 Hz. Programming strategies were individualized and could include parameter modification in response to insufficient analgesia, habituation, or recurrence of symptoms.

Initial stimulation programming was performed after postoperative recovery, typically within the first weeks following implantation. Patients were followed longitudinally in dedicated neuromodulation outpatient clinics. Reprogramming sessions were performed as clinically indicated, including in cases of insufficient analgesic response, recurrence of pain symptoms, or changes in stimulation efficacy over time. The frequency and timing of reprogramming sessions were documented as part of routine clinical care.

For this study, frequent reprogramming was predefined as ≥6 reprogramming sessions during follow-up. The primary outcome measure was pain intensity assessed using a numerical rating scale (NRS) or visual analog scale (VAS). All scores were analyzed on a unified 0–10 metric. When VAS was recorded on a 0–100 scale, values were converted to a 0–10 scale by dividing by 10. Pain intensity was recorded at baseline (preoperative), during early postoperative follow-up, and at subsequent long-term follow-up visits. Secondary outcome measures included: best achieved pain response, defined as the lowest documented NRS/VAS score during follow-up; responder status, defined according to pain intensity at the last available follow-up (responder ≥ 50% reduction; partial responder 30–49%; non-responder < 30%); loss of efficacy over time, defined as an increase of ≥2 NRS/VAS points compared to the best achieved score during follow-up; clinically relevant OFF worsening, defined as an increase of ≥2 NRS/VAS points or recurrence of typical neuropathic pain symptoms during stimulator deactivation (intentional OFF testing or battery depletion); changes in analgesic medication (dose reduction, discontinuation, no change, or increase), when documented; device-related outcomes, including stimulation reprogramming, IPG replacement, lead revision, or explantation; and adverse events and complications, including infection, lead migration, or clinically relevant stimulation-related side effects. OFF periods were not assessed using a standardized prospective protocol. Instead, OFF-related observations were derived from routine clinical practice and documented in medical records. Temporary stimulation deactivation occurred either intentionally, for clinical evaluation of stimulation efficacy, or unintentionally, most commonly due to battery depletion. The duration of stimulation interruption and timing of pain reassessment were not standardized and varied between patients. For the purpose of this study, OFF-related changes were considered only when clearly documented in the clinical record and consistent with the patient’s typical neuropathic pain pattern.

Adverse events were assessed retrospectively based on clinical documentation. Clinically relevant adverse events were defined as stimulation-related or hardware-related complications requiring surgical revision, hospitalization, or device explantation. Minor or transient adverse effects were not systematically captured due to the retrospective study design. Clinical data were extracted at predefined time points when available, including baseline, early postoperative follow-up, intermediate follow-up (approximately 6–12 months), and the most recent long-term follow-up visit. For patients with extended follow-up, longitudinal pain trajectories were reconstructed based on all available documented assessments. Follow-up duration was calculated from the date of MCS implantation to the date of the most recent documented clinical evaluation or death, when applicable.

Statistical Analysis

All statistical analyses were conducted using MedCalc Statistical Software, version 12.5.0 (MedCalc Software Ltd., Ostend, Belgium). Descriptive statistical methods were employed to summarize patient demographics, pain characteristics, and clinical outcomes. Continuous variables are reported as mean ± standard deviation or median with interquartile range, depending on data distribution. Categorical variables are presented as absolute counts and percentages. Given the retrospective design, limited sample size, and exploratory nature of this cohort analysis, the study was not powered for formal hypothesis testing. Accordingly, no inferential statistical tests were performed. Instead, longitudinal changes in pain intensity and device-related outcomes were evaluated descriptively to provide a transparent representation of individual and cohort-level trends over extended follow-up.

## 3. Results

Ten patients with refractory neuropathic orofacial pain treated with epidural MCS were included in this retrospective cohort. The cohort consisted of six women (60%) and four men (40%), with a mean age of 61.5 ± 8.6 years at the time of implantation (range 46–74 years). Pain etiology was heterogeneous: post-surgical neuropathic pain in five patients (50%), traumatic neuropathic pain in two (20%), cerebrovascular injury-related pain in two (20%), and post-herpetic neuralgia in one patient (10%).

All patients experienced chronic neuropathic pain prior to implantation, with a mean pain duration of 6.1 ± 6.2 years (range 2–21 years). Pain was unilateral in all cases, affecting the left side in seven patients (70%) and the right side in three (30%). Trigeminal involvement was classified as mixed (V1/V2/V3) in all patients.

Baseline pain intensity was severe across the cohort, with a mean NRS/VAS score of 8.8 ± 0.4 (0–10 scale). Baseline demographic and clinical characteristics are summarized in Table 1. Detailed individual patient characteristics and device-related parameters are provided in Supplementary Table S1.

All patients underwent epidural implantation of a paddle electrode over the primary motor cortex contralateral to the dominant pain side, targeting the facial representation area. No intraoperative complications were reported. All patients required stimulation reprogramming during follow-up. The median number of reprogramming sessions per patient was 9 (range 4–25). Across the cohort, a total of 113 reprogramming sessions were performed over a cumulative follow-up of 76 patient-years, corresponding to an average of approximately 1.5 sessions per patient-year. Frequent reprogramming, as predefined in the Methods section, occurred in eight patients (80%). A change in stimulation paradigm over time was documented in six patients (60%). The mean documented follow-up duration was 7.6 ± 6.3 years (range 2–22 years). Seven patients (70%) were followed for ≥5 years, while three patients (30%) had follow-up durations between 2 and 5 years. The mean best achieved pain score during follow-up was 4.6 ± 0.8. At the last documented follow-up, the mean pain score was 5.6 ± 0.9, remaining lower than baseline across the cohort.

**Figure 1:**
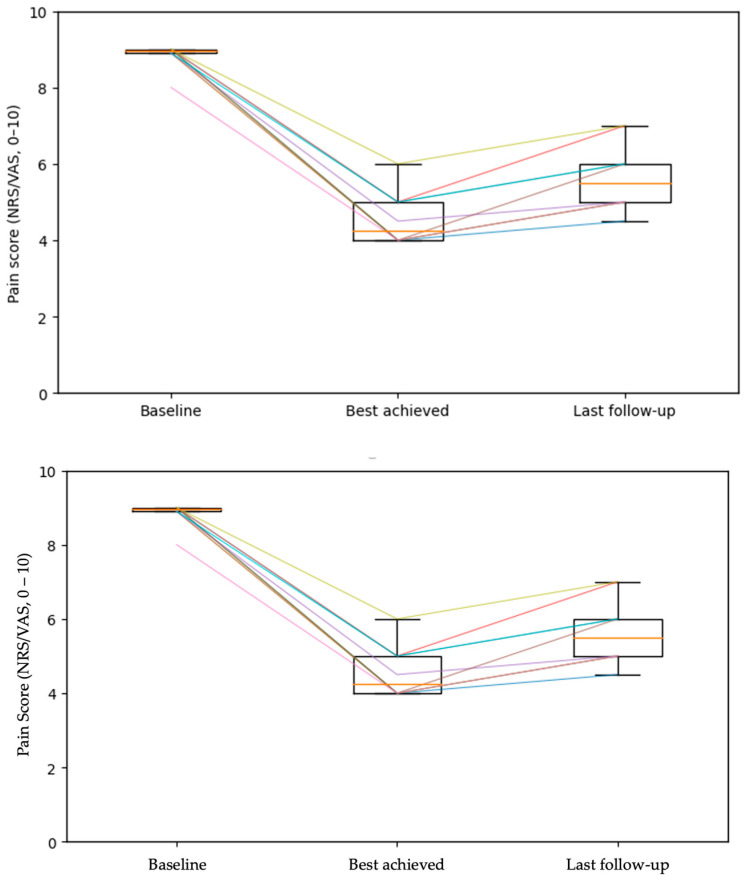
Pain intensity over time in patients treated with MCS. Pain intensity scores (NRS/VAS, 0–10 scale) are shown at baseline, best achieved response during follow-up, and at the last documented follow-up. Thin lines represent individual patient trajectories, illustrating within-patient changes over time. Boxplots summarize the group-level distribution at each time point, with boxes indicating the interquartile range, horizontal lines representing the median, and whiskers denoting the observed range. Pain intensity decreased markedly at the best achieved response and remained lower than baseline at the last follow-up.

Responder status was determined at the last available follow-up. Based on the prespecified threshold of ≥50% pain reduction from baseline, no patients met criteria for responder status at the last available follow-up. Eight patients (80%) were classified as partial responders (30–49% reduction), while two patients (20%) met criteria for non-response (<30% reduction). All response categories were cross-checked against patient-level data to ensure consistency across the main text, Table 2, and Supplementary Table S1. Loss of efficacy over time was documented in three patients (30%), whereas seven patients (70%) maintained sustained clinical benefit throughout follow-up. When documented in the clinical record, analgesic therapy at the last follow-up was often reduced or simplified, most commonly involving dose reduction and/or discontinuation of anticonvulsants and antidepressants. These observations were based on available clinical documentation and reflect long-term treatment patterns rather than short-term adjustments. Pain intensity trajectory is illustrated in Figure 1.

**Figure 2:**
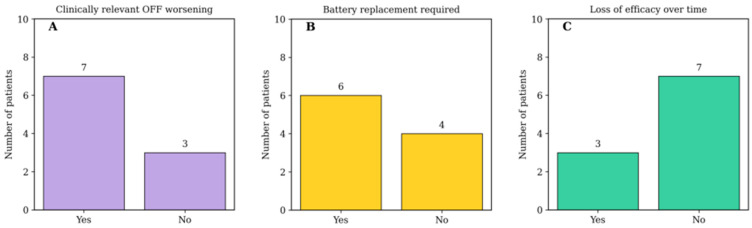
Long-term stimulation dependency and device-related outcomes in patients treated with MCS. Bar charts illustrate key long-term stimulation and device-related outcomes: (**A**) clinically relevant worsening of pain during stimulator OFF periods; (**B**) requirement for battery replacement during follow-up; and (**C**) documented loss of analgesic efficacy over time. The *y*-axis represents the number of patients in each category. Numerical values above each bar indicate absolute patient counts.

Clinically relevant worsening of pain during stimulator OFF periods was documented in seven patients (70%). In three patients (30%), no clear worsening during OFF periods was observed. Battery replacement was required in six patients (60%). Among these patients, the mean number of replacements was 1.7, with time to first replacement ranging from 1 to 6 years. Initial systems were predominantly non-rechargeable, while patients requiring replacement were transitioned to rechargeable implantable pulse generators in accordance with evolving clinical practice. No hardware-related complications were documented. At the last available follow-up assessment (or last assessment before death), all stimulation systems remained active, and no explantations were performed due to adverse events or lack of efficacy. Comprehensive long-term clinical and device-related outcomes are summarized in Table 2, and key categorical outcomes are illustrated in Figure 2. In addition, detailed individual device evolution and programming characteristics are presented in Supplementary Table S1.

No clinically significant stimulation-related adverse effects requiring surgical revision, device removal, or hospitalization were recorded during follow-up. Minor or transient adverse effects may not have been systematically captured due to the retrospective nature of the study. Three patients died during extended follow-up due to causes unrelated to MCS therapy. These patients were included in the analysis up to their last documented clinical assessment in accordance with the predefined study methodology.

## 4. Discussion

The present study provides additional long-term real-world data on epidural MCS in patients with refractory neuropathic orofacial pain. Despite more than three decades of clinical use since the initial reports [8,9], the evidence base remains dominated by small retrospective series with heterogeneous outcome definitions and limited reporting of long-term stimulation dependency and device management [18,19,20]. More recent syntheses in chronic neuropathic orofacial pain underline both the heterogeneity of indications and the need for standardized reporting and longer follow-up when evaluating invasive motor cortex stimulation [14]. In that context, our cohort, characterized by extended documented follow-up and systematic capture of clinically relevant OFF effects, adds longitudinal detail that complements prior literature rather than attempting to redefine it.

In line with prior clinical experience, MCS was associated with clinically meaningful pain reduction in most patients, with a decrease from severe baseline pain to moderate levels at the best achieved response [12,13,17]. At the last documented follow-up, pain scores remained lower than baseline at the cohort level, although loss of efficacy over time was documented in a subset of patients. This pattern, initial improvement followed by partial attenuation in some cases, has been discussed in long-term series and reviews as a realistic feature of chronic neuromodulation rather than an exception [15,18,19]. Importantly, responder classification in our study was anchored to the last available follow-up rather than peak response, providing a conservative durability estimate and helping harmonize reporting across time points.

A clinically important observation was OFF-related worsening in 70% of patients. OFF–ON comparisons are central to interpreting neuromodulation effects, yet they are inconsistently reported in older MCS series. In the present study, OFF-related observations were derived from routine clinical practice rather than a standardized prospective protocol, and included both intentional temporary deactivation of stimulation and unintentional interruptions, most commonly related to battery depletion. While this heterogeneity limits formal comparison across patients, these real-world observations provide clinically relevant insight into the functional role of ongoing stimulation. Although this finding cannot exclude longer-term neuroplastic contributions, it is consistent with the clinical observation that ongoing stimulation is often required to maintain perceived benefit in routine practice. This observation is congruent with controlled evidence indicating measurable differences between active and sham stimulation conditions in invasive MCS [16]. It also aligns with broader neuromodulation literature that frames such therapies as device-dependent, adjustable interventions rather than one-time curative procedures [22].

Although OFF-related worsening is consistent with a stimulation-dependent effect, these observations are based on non-standardized clinical assessments and do not allow definitive mechanistic conclusions.

Mechanistically, MCS is increasingly conceptualized as modulation of distributed pain-processing networks rather than purely focal somatotopic effects. In this framework, emerging evidence suggests that the cervicotrigeminal complex may act as an important integrative structure in head and neck neuropathic pain, functionally linking trigeminal and upper cervical nociceptive pathways within broader thalamo-cortical and brainstem circuits [23]. Given the anatomical and functional connectivity between the motor cortex, thalamus, periaqueductal gray, and brainstem nuclei, it is plausible that MCS may influence descending modulatory systems converging on trigeminal and cervicomedullary processing levels, although these interpretations remain inferential and should be interpreted with caution in the context of a small retrospective cohort. Prior work has demonstrated the involvement of cortico-thalamic and cortico-limbic circuits in motor cortex-mediated analgesia [10,11], while more recent mechanistic syntheses frame MCS-related analgesia as an emergent network phenomenon involving multiple hierarchical levels of the neuroaxis [24]. Within such a distributed network model, the dependence on active stimulation observed in our cohort is compatible with the hypothesis that sustained neuromodulatory input may be required to maintain a rebalanced functional state across interconnected cortical and subcortical structures. However, these mechanistic considerations are intended to provide a conceptual framework for interpretation and should not be considered as direct evidence derived from the present dataset.

The longitudinal reprogramming burden in our cohort, universal reprogramming, and frequent reprogramming in the majority of patients, reinforce the practical reality that long-term outcomes are shaped not only by surgical targeting but also by iterative programming optimization. Contemporary reviews emphasize variability of response rates and the need for adaptive programming strategies over time [15].

In addition, selection pathways using non-invasive motor cortex stimulation as a predictor of invasive MCS response have been summarized in recent systematic work [25], supporting the broader movement toward improved preoperative stratification, although this was not a focus of the present retrospective cohort.

Device management further highlights MCS as a chronic neuromodulatory therapy: battery replacement occurred in 60% of patients, reflecting prolonged system use rather than device failure. No hardware infections, lead migrations, or clinically relevant stimulation-related adverse effects were documented. This is consistent with the general view that, in experienced centers, invasive cortical stimulation can have an acceptable long-term safety profile, while still requiring realistic counseling about device maintenance and follow-up [15,26]. Recent perspective pieces also emphasize conceptual refinements in terminology and targeting, framing this approach more broadly as precentral cortex stimulation rather than strictly “motor cortex” stimulation, reflecting evolving understanding of stimulation fields and network anatomy [27].

Although our cohort focused on neuropathic orofacial pain, MCS has been applied across neuropathic pain syndromes, including post-stroke central pain, brachial plexus injury, spinal cord injury-related pain, phantom limb pain, and complex regional pain syndrome [12,13,15,18,19]. However, the overall evidence remains limited by heterogeneity in etiology, phenotyping, and outcome definitions, as underscored by modern systematic analyses in orofacial neuropathic pain [14]. Therefore, while our data support feasibility and long-term management characteristics in a real-world cohort, they should be interpreted as complementary to the broader literature rather than definitive for patient selection or comparative efficacy.

Several limitations should be acknowledged. The retrospective design and small sample size limit generalizability and preclude inferential statistics. Given the rarity of carefully phenotyped, surgically treated neuropathic orofacial pain cohorts undergoing invasive motor cortex stimulation, small sample sizes remain a structural limitation of the field. The present study should therefore be interpreted as a detailed longitudinal cohort description rather than a definitive efficacy trial. Pain assessment relied primarily on NRS/VAS scores, which, while widely used in neuropathic pain research and clinical practice [1,2], do not capture multidimensional pain domains. Multidimensional outcome measures, including quality of life, functional status, sleep, and validated neuropathic pain questionnaires, were not systematically available due to the retrospective design and extended inclusion period, limiting assessment of broader clinical impact beyond pain intensity. Follow-up intervals and programming strategies were not standardized, reflecting real-world clinical care, but limiting cross-patient comparability. The single-center design may introduce referral bias, and objective neurophysiological correlations were not available. Moreover, as this cohort was treated at a tertiary referral center with dedicated expertise in stereotactic and functional neurosurgery, the findings may not be directly generalizable to centers with different patient selection pathways or neuromodulation experience. The absence of a control group or sham stimulation phase limits causal inference regarding the magnitude of the observed effect. Device technology also evolved during the study period, including the transition from non-rechargeable to rechargeable systems, which may have influenced battery longevity and programming strategies. This reflects real-world clinical practice but introduces additional heterogeneity when interpreting long-term outcomes. Adverse event reporting was limited to clinically significant events documented in medical records, and minor or transient effects may have been underreported. In addition, detailed longitudinal pharmacological data, including exact dosing and timing of treatment adjustments, were not uniformly available across all patients, which precludes detailed analysis of medication trajectories and limits interpretation of treatment-related changes beyond qualitative observations. Nevertheless, extended follow-up, harmonized response definitions, systematic OFF documentation, and detailed reporting of device management provide clinically useful longitudinal insight and help address reporting gaps highlighted in recent reviews [14,15].

## 5. Conclusions

This long-term single-center cohort study suggests that epidural MCS is associated with sustained and clinically meaningful pain reduction in carefully selected patients with refractory neuropathic orofacial pain. The findings are consistent with the interpretation of MCS as a chronic neuromodulatory therapy requiring ongoing stimulation, individualized programming, structured long-term follow-up, and device management.

The systematic documentation of pain worsening during stimulator OFF periods in a substantial proportion of patients is consistent with dependence on ongoing stimulation in routine clinical practice. However, the present data do not allow definitive mechanistic conclusions to be drawn and should be interpreted within the limitations of a retrospective design.

The need for repeated reprogramming and device maintenance further emphasizes that long-term outcomes depend not only on surgical targeting but also on adaptive therapy management over time. While our cohort focused on neuropathic orofacial pain, the observations may be relevant to other refractory neuropathic pain conditions characterized by central network dysfunction, although this requires confirmation in larger and more diverse populations.

Future prospective, ideally multicenter studies incorporating standardized outcome measures and mechanistic biomarkers are warranted to better define patient selection criteria, optimize stimulation strategies, and clarify the long-term trajectory of MCS therapy.

## Figures and Tables

**Table 1:** Baseline demographic and clinical characteristics of patients treated with MCS.

Variable	Value
Number of patients	10
Sex (female/male)	6 (60%)/4 (40%)
Age at implantation, years	61.5 ± 8.6 (range 46–74)
Etiology	Post-surgical 5 (50%); Traumatic 2 (20%); CVI-related 2 (20%); Post-herpetic 1 (10%)
Pain duration before MCS, years	6.1 ± 6.2 (range 2–21)
Pain laterality	Left 7 (70%); Right 3 (30%)
Trigeminal distribution	Mixed V1/V2/V3: 10 (100%)
Baseline pain score (NRS/VAS, 0–10)	8.8 ± 0.4

**Table 2:** Long-term clinical and device-related outcomes in patients treated with MCS.

Variable	Value
Best achieved pain score (NRS/VAS, 0–10)	4.6 ± 0.8
Pain score at last follow-up (NRS/VAS, 0–10)	5.6 ± 0.9
Follow-up duration, years	7.6 ± 6.3 (range 2–22)
Follow-up ≥ 5 years	7 (70%)
Responders at last follow-up (≥50% reduction)	0 (0%)
Partial responders at last follow-up (30–49% reduction)	8 (80%)
Non-responders at last follow-up (<30% reduction)	2 (20%)
Loss of efficacy over time	3 (30%)
Clinically relevant OFF worsening	7 (70%)
Reprogramming required	10 (100%)
Reprogramming sessions per patient	median 9 (range 4–25)
Frequent reprogramming (predefined)	8 (80%)
Change in stimulation paradigm over time	6 (60%)
Battery replacement required	6 (60%)
Battery replacements (among those requiring replacement)	mean 1.7
Time to first battery replacement, years	1–6
Hardware-related complications	0
Device explantation	0
Stimulation-related adverse effects	0
Death during follow-up	3 (30%)

## Data Availability

The datasets generated and analyzed during the current study are not publicly available due to ethical and privacy restrictions, but are available from the corresponding author on reasonable request and subject to institutional and ethical approval.

## Supplementary Materials

The following supporting information can be downloaded at https://www.mdpi.com/article/10.3390/life16040651/s1. Table S1: Individual Patient Characteristics and Device-Related Data.

## Abbreviations

The following abbreviations are used in this manuscript:

MCS	Motor cortex stimulation
NRS	Numerical rating scale
VAS	Visual analog scale
IPG	Implantable pulse generator
IASP	International Association for the Study of Pain
CVI	Cerebrovascular injury

## References

[1] Treede R.D., Jensen T.S., Campbell J.N., Cruccu G., Dostrovsky J.O., Griffin J.W., Hansson P., Hughes R., Nurmikko T., Serra J. (2008). Neuropathic pain: Redefinition and a grading system for clinical and research purposes. Neurology.

[2] Raja S.N., Carr D.B., Cohen M., Finnerup N.B., Flor H., Gibson S., Keefe F.J., Mogil J.S., Ringkamp M., Sluka K.A. (2020). The revised International Association for the Study of Pain definition of pain: Concepts, challenges, and compromises. Pain.

[3] Scholz J., Finnerup N.B., Attal N., Aziz Q., Baron R., Bennett M.I., Benoliel R., Cohen M., Cruccu G., Davis K.D. (2019). The IASP classification of chronic pain for ICD-11: Chronic neuropathic pain. Pain.

[4] Benoliel R., Svensson P., Heir G.M., Sirois D., Zakrzewska J., Oke-Nwosu J., Torres S.R., Greenberg M.S., Klasser G.D., Katz J. (2011). Persistent orofacial muscle pain. Oral Dis..

[5] Zakrzewska J.M., Linskey M.E. (2014). Trigeminal neuralgia. BMJ.

[6] Baad-Hansen L. (2008). Atypical odontalgia-pathophysiology and clinical management. J. Oral Rehabil..

[7] Cruccu G., Finnerup N.B., Jensen T.S., Scholz J., Sindou M., Svensson P., Treede R.D., Zakrzewska J.M., Nurmikko T. (2016). Trigeminal neuralgia: New classification and diagnostic grading for practice and research. Neurology.

[8] Tsubokawa T., Katayama Y., Yamamoto T., Hirayama T., Koyama S. (1991). Chronic motor cortex stimulation for the treatment of central pain. Acta Neurochirurgica Supplementum.

[9] Tsubokawa T., Katayama Y., Yamamoto T., Hirayama T., Koyama S. (1993). Chronic motor cortex stimulation in patients with thalamic pain. J. Neurosurg..

[10] Peyron R., Faillenot I., Mertens P., Laurent B., Garcia-Larrea L. (2007). Motor cortex stimulation in neuropathic pain. Correlations between analgesic effect and hemodynamic changes in the brain. A PET study. Neuroimage.

[11] Garcia-Larrea L., Peyron R. (2007). Motor cortex stimulation for neuropathic pain: From phenomenology to mechanisms. Neuroimage.

[12] Nuti C., Peyron R., Garcia-Larrea L., Brunon J., Laurent B., Sindou M., Mertens P. (2005). Motor cortex stimulation for refractory neuropathic pain: Four-year outcome and predictors of efficacy. Pain.

[13] Lazorthes Y., Sol J.C., Fowo S., Roux F.E., Verdié J.C. (2007). Motor cortex stimulation for neuropathic pain. Acta Neurochirurgica Supplementum.

[14] Henssen D., Kurt E., van Walsum A.V.C., Kozicz T., van Dongen R., Bartels R. (2020). Motor cortex stimulation in chronic neuropathic orofacial pain syndromes: A systematic review and meta-analysis. Sci. Rep..

[15] Ramos-Fresnedo A., Perez-Vega C., Domingo R.A., Cheshire W.P., Middlebrooks E.H., Grewal S.S. (2022). Motor Cortex Stimulation for Pain: A Narrative Review of Indications, Techniques, and Outcomes. Neuromodulation.

[16] Hamani C., Fonoff E.T., Parravano D.C., Silva V.A., Galhardoni R., Monaco B.A., Navarro J., Yeng L.T., Teixeira M.J., de Andrade D.C. (2021). Motor cortex stimulation for chronic neuropathic pain: Results of a double-blind randomized study. Brain.

[17] Nguyen J.P., Lefaucher J.P., Le Guerinel C., Eizenbaum J.F., Nakano N., Carpentier A., Brugières P., Pollin B., Rostaing S., Keravel Y. (2000). Motor cortex stimulation in the treatment of central and neuropathic pain. Arch. Med. Res..

[18] Rasche D., Ruppolt M., Stippich C., Unterberg A., Tronnier V.M. (2006). Motor cortex stimulation for long-term relief of chronic neuropathic pain: A 10-year experience. Pain.

[19] Carroll D., Joint C., Maartens N., Shlugman D., Stein J., Aziz T.Z. (2000). Motor cortex stimulation for chronic neuropathic pain: A preliminary study of 10 cases. Pain.

[20] Boccard S.G., Pereira E.A., Moir L., Aziz T.Z., Green A.L. (2013). Long-term outcomes of deep brain stimulation for neuropathic pain. Neurosurgery.

[21] Woolf C.J. (2011). Central sensitization: Implications for the diagnosis and treatment of pain. Pain.

[22] Fisher L.E., Lempka S.F. (2023). Neurotechnology for Pain. Annu. Rev. Biomed. Eng..

[23] Raguž M., Tarle M., Hat K., Salarić I., Marčinković P., Bičanić I., Lazić Mosler E., Lukšić I., Marinović T., Chudy D. (2025). Refractory Neuropathic Pain in the Head and Neck: Neuroanatomical and Clinical Significance of the Cervicotrigeminal Complex. Life.

[24] DosSantos M.F., Ferreira N., Toback R.L., Carvalho A.C., DaSilva A.F. (2016). Potential Mechanisms Supporting the Value of Motor Cortex Stimulation to Treat Chronic Pain Syndromes. Front. Neurosci..

[25] Gatzinsky K., Bergh C., Liljegren A., Silander H., Samuelsson J., Svanberg T., Samuelsson O. (2020). Repetitive transcranial magnetic stimulation of the primary motor cortex in management of chronic neuropathic pain: A systematic review. Scand. J. Pain.

[26] Velasco F., Argüelles C., Carrillo-Ruiz J.D., Castro G., Velasco A.L., Jiménez F., Velasco M. (2008). Efficacy of motor cortex stimulation in the treatment of neuropathic pain: A randomized double-blind trial. J. Neurosurg..

[27] Lefaucheur J.P., Nguyen J.P., Hodaj H., Sindou M., Bardel B. (2025). Cortical stimulation to treat chronic pain: No longer call it “motor cortex stimulation” but “precentral cortex stimulation”. Brain Stimul..

