# Supplementary material for: Long-Term Outcomes of Epidural Motor Cortex Stimulation for Refractory Chronic Neuropathic Orofacial Pain
Source: Life (Basel). 2026 Apr 12;16(4):651. doi: 10.3390/life16040651 (PMC13117579; doi:10.3390/life16040651)
Supplement: Supplementary file 1 [file life-16-00651-s001.zip › life-4216636-supplementary.pdf]

Supplementary Table S1. Individual Patient Characteristics and Device-Related Data.

No	Sex	Age	Etiology	Pain duration (y)	Baseline NRS	Best NRS	Last NRS	FU (y)	Responder	Loss of efficacy	OFF worsening	Reprog (n)	Frequent reprog	Initial electrode	Initial IPG	Current electrode	Current IPG	System status at last contact
**1**	M	59	CVI	4	8.9	4	4.5	6	Partial	No	Yes	17	Yes	SureScan 5-6-5	Vanta	SureScan 5-6-5	Vanta	Active*
**2**	M	64	CVI	2	8.9	4	5	2	Partial	No	No	4	No	SureScan 5-6-5	Vanta	SureScan 5-6-5	Inceptiv (R)	Active
**3**	F	46	Trauma	2	9	4	5	22	Partial	No	Yes	25	Yes	Resume TL	PrimeAdvanced	SureScan 5-6-5	Inceptiv (R)	Active
**4**	M	63	Trauma	21	9	5	7	15	No	Yes	Yes	21	Yes	Resume TL	PrimeAdvanced	SureScan 5-6-5	Inceptiv (R)	Active*
**5**	F	65	Surgery	6	8.9	4.5	5	7	Partial	No	Yes	8	Yes	SureScan 5-6-5	Vanta	SureScan 5-6-5	Vanta	Active
**6**	F	47	Surgery	5	9	4	6	10	Partial	Yes	Yes	11	Yes	Resume TL	PrimeAdvanced	SureScan 5-6-5	Inceptiv (R)	Active
**7**	F	67	Surgery	2	8	4	5	2	Partial	No	No	5	No	SureScan 5-6-5	Vanta	SureScan 5-6-5	Inceptiv (R)	Active
**8**	F	74	Herpes	5	9	5	6	2	Partial	No	Yes	6	Yes	SureScan 5-6-5	Vanta	SureScan 5-6-5	Vanta	Active
**9**	F	68	Surgery	10	9	6	7	3	No	Yes	No	6	Yes	SureScan 5-6-5	Vanta	SureScan 5-6-5	Vanta	Active
**10**	M	62	Surgery	4	8.9	5	6	7	Partial	No	Yes	10	Yes	SureScan 5-6-5	Vanta	SureScan 5-6-5	Inceptiv (R)	Active*

FU, follow-up; IPG, implantable pulse generator; NRS, Numerical Rating Scale. Responder is defined as a≥50% reduction in pain intensity at last follow-up compared with baseline. A partial responder is defined as a 30–49% reduction in pain intensity at last follow-up. Loss of efficacy is defined as an increase of ≥2 points in NRS/VAS compared to the best achieved pain score during follow-up. Clinically relevant OFF worsening is defined as a≥2-point increase in NRS during device deactivation or battery depletion. Frequent reprogramming is defined as ≥6 reprogramming sessions during follow-up. In three patients with the longest follow-up (22, 15, and 10 years), the initial system consisted of a Medtronic Resume TL paddle electrode connected to a Medtronic PrimeAdvanced IPG; during long-term follow-up, a complete system revision was performed with implantation of a Medtronic SureScan Specify™ 5-6-5 paddle electrode and upgrade to a Medtronic Inceptiv IPG. All other patients initially received a SureScan Specify™ 5-6-5 electrode connected to a Medtronic Vanta IPG; upon battery depletion, the IPG was replaced with a Medtronic Inceptiv rechargeable device. System status "Active" indicates that stimulation was ongoing until the patient's death.*
